# Drug-microbiota interactions and treatment response: Relevance to rheumatoid arthritis

**DOI:** 10.3934/microbiol.2018.4.642

**Published:** 2018-10-26

**Authors:** Ellie Sayers, Alex MacGregor, Simon R. Carding

**Affiliations:** 1 Gut Health and Food Safety Programme, Quadram Institute Bioscience, Norwich, Norfolk, NR4 7UA, UK; 2 Norwich Medical School, Faculty of Medicine and Health Sciences, University of East Anglia, Norwich, Norfolk, NR4 7TJ, UK

**Keywords:** intestinal microbiota, metabolism, methotrexate, drug response

## Abstract

Knowledge about associations between changes in the structure and/or function of intestinal microbes (the microbiota) and the pathogenesis of various diseases is expanding. However, interactions between the intestinal microbiota and different pharmaceuticals and the impact of these on responses to treatment are less well studied. Several mechanisms are known by which drug-microbiota interactions can influence drug bioavailability, efficacy, and/or toxicity. This includes direct activation or inactivation of drugs by microbial enzymes which can enhance or reduce drug effectiveness. The extensive metabolic capabilities of the intestinal microbiota make it a hotspot for drug modification. However, drugs can also influence the microbiota profoundly and change the outcome of interactions with the host. Additionally, individual microbiota signatures are unique, leading to substantial variation in host responses to particular drugs. In this review, we describe several known and emerging examples of how drug-microbiota interactions influence the responses of patients to treatment for various diseases, including inflammatory bowel disease, type 2 diabetes and cancer. Focussing on rheumatoid arthritis (RA), a chronic inflammatory disease of the joints which has been linked with microbial dysbiosis, we propose mechanisms by which the intestinal microbiota may affect responses to treatment with methotrexate which are highly variable. Furthering our knowledge of this subject will eventually lead to the adoption of new treatment strategies incorporating microbiota signatures to predict or improve treatment outcomes.

## 1. Introduction

With the advent of high-throughput next-generation DNA sequencing (NGS), our knowledge about the characteristics and capabilities of the human intestinal microbiota in relation to disease etiology and progression, has expanded enormously [1]. Microbial dysbiosis; an imbalance in the compositional and/or functional profiles of the intestinal microbiota, has been linked to several conditions including inflammatory bowel disease (IBD), obesity, diabetes, and rheumatoid arthritis (RA) [1]–[5]. Thus far, connections between microbial profiles and disease states have been associative. The underlying mechanisms by which resident microbes affect the etiology and progression of disease are still largely unknown. Understanding the roles that these microbes play in both the development of disease and in determining the efficacy of various treatments could facilitate the development of new treatments and interventions with the potential to improve long-term disease outcomes.

The intestinal microbiota makes up the largest and most diverse community of resident microbes in the human body [6]. It has a vast array of metabolic capabilities that is more diverse than the enzyme-coding capacity of the human genome. However, the role of the intestinal microbiota in the metabolism of xenobiotics, such as pharmaceuticals, and its subsequent effect on human physiology and treatment responses is little understood. Inter-individual microbiota variability is the largest contributor of variability in microbial community composition; although a core set of microbes and microbial functions are shared among individuals [7]. Host genetics also has a direct impact on interactions between microbes. Furthermore, microbial metabolism of xenobiotics is dependent on the specific microenvironment, including the presence of nutrients or cofactors that are either derived from exogenous sources or provided by other microorganisms in the community. Despite this complexity studies on the microbial metabolism of xenobiotics, particularly pharmaceuticals, have clearly demonstrated that the intestinal microbiota has a strong effect on clinical responses to treatment and drug toxicity [8]. Any orally-administered pharmaceuticals that are not absorbed in the upper GI-tract will eventually come into contact with the intestinal microbiota. Parenterally administered drugs may also contact the intestinal microbiota if they are excreted in bile, a process known as enterohepatic recirculation.

A good example of the importance of interactions between drugs and resident microbial populations can be seen for the vaginal microbiota where the relative abundance of particular bacteria determines the efficacy of the antiretroviral drug, tenofovir. Typically, species in the genus *Lactobacillus* dominate the vaginal microbiota. However, in around 39% of women taking part in a trial investigating the clinical efficacy of tenofovir, *Gardnerella vaginalis* replaced *Lactobacillus* spp. as the dominant species [9]. In those women with a *G. vaginalis*-dominated vaginal microbiota, concentrations of tenofovir were reduced, possibly because *G. vaginalis* rapidly metabolizes tenofovir before it is taken up by human cells [9]. A more detailed understanding of the mechanisms driving microbe-drug interactions may therefore be beneficial in the prevention and treatment of a number of diseases where the efficacy of drugs and topical treatments is highly variable. It is also possible that some treatment regimens could be improved by considering individual patients' microbiotas.

## 2. Mechanisms by which intestinal microbiota influence drug bioavailability and efficacy

**Figure 1.**
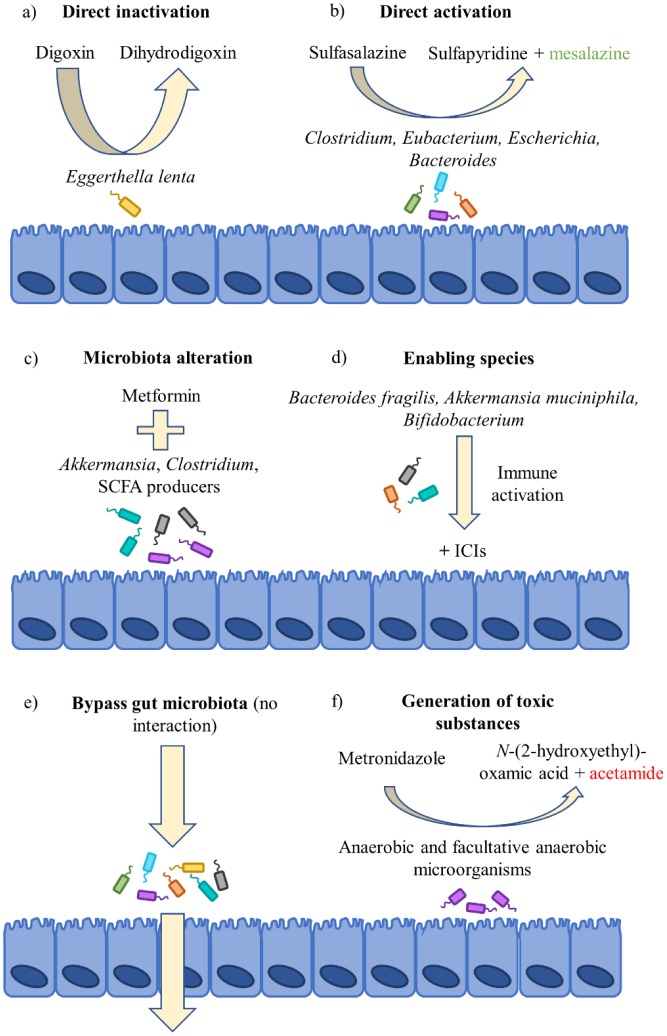
Mechanisms for drug-microbiota interactions. There are several known mechanisms for drug-microbiota interaction that may affect treatment outcomes. Of course, it is not always the case that there will be interactions. For example, some drugs may bypass the intestinal microbiota altogether (e). However, others will be enzymatically activated (b) or inactivated (a) by certain microbes or be converted into potentially toxic substances (f). More recently, the composition of the microbiota has been linked to treatment outcomes, either in association with taking a drug (c) or in response to the existence of certain microbes prior to its use (d). ICI = immune checkpoint inhibitor; SCFA = short chain fatty acid.

Several known mechanisms exist by which the intestinal microbiota can either directly, or indirectly, change the bioavailability and/or efficacy of drugs (Figure 1). Microbial and host metabolism of xenobiotics differ with respect to the type of metabolic reactions they employ. For example, microbes predominantly use reductive and hydrolytic reactions whereas their human hosts predominantly use oxidation and conjugation [8]. Orthologous enzymes for basic processes do exist between microbiota and their hosts, although the microbiota has access to a much larger library of metabolic processes than the host. This is due to the extensive diversity of species within the microbiota, which has been exploited to generate various prodrugs (e.g. sulfasalazine and metronidazole) that are activated by microbial enzymes. Many microbial processes benefit the host, for example, by converting dietary fibre to short-chain fatty acids (SCFAs) that are anti-inflammatory and provide energy to intestinal epithelial cells [10]. In contrast, microbial β-glucuronidase activity directed at the cancer drug irinotecan induces severe toxicity in the form of diarrhea which is dose-limiting and impacts on treatment efficacy [11].

### 2.1. Inactivation of digoxin by Eggerthella lenta

Digoxin is a toxin derived from plants and is used to treat congestive heart failure and arrhythmia. In approximately 10% of patients the bioavailability and efficacy of digoxin is greatly reduced, and is replaced by an increase in cardioinactive, reduced metabolites of digoxin [12]. In the early 1980s it was shown that this lack of bioavailability could be restored by co-administration of antibiotics that depleted the intestinal microbiota [12]. Later, the same group identified the digoxin-inactivating properties of a single species of bacterium, *Eggerthella lenta*, that was present in the intestinal microbiota; this is the only known organism capable of reducing digoxin to dihydrodigoxin, thereby rendering it inactive [13]. *E. lenta* is a common member of the intestinal microbiota, but its presence alone is not sufficient to predict digoxin inactivation; only specific strains containing the *cgr1* and *cgr2* genes can reduce digoxin to dihydrodigoxin and the presence of these genes can be used as a marker for potential clinical non-response [14]. Interestingly, the *cgr1*/*cgr2* operon is inhibited by the presence of the amino acid, arginine. In mice given digoxin and fed diets high in protein the concentrations of digoxin in the serum and urine were increased. This suggests that dietary supplementation with protein (or arginine) may increase digoxin bioavailability by preventing its reduction by *E. lenta* [14]. Supplementation with arginine may be useful to increase the bioavailability and efficacy of digoxin in people with *E. lenta* carrying *cgr1*/*cgr2*.

### 2.2. Prodrug activation and toxic by-products

A prodrug is an inactive medication or compound that, after administration, is converted by enzymatic action into a pharmacologically active drug. Sulfasalazine (SSZ) is a prodrug used to treat inflammation in patients with IBD or RA. It is converted into an active form by the intestinal microbiota via azo reductase, an enzyme that cleaves SSZ into sulfapyridine (a potentially toxic by-product) and 5-aminosalicyclic acid (5-ASA/mesalazine) which is the main anti-inflammatory component. Azo reductases are produced by a variety of intestinal bacteria, including species of *Clostridium*, *Eubacterium* and *Bacteroides* [15],[16]. The majority of 5-ASA is retained in the colon undergoing enterohepatic recirculation and is eventually excreted in feces, making it ideal for the treatment of IBD [17]. The abundance of azo reductase enzymes in the intestinal microbiota has not been formally measured but could potentially have an impact on treatment responses to this drug. Although one product of microbial metabolism of SSZ is active as an anti-inflammatory, the other (sulfapyridine) has an antimicrobial effect and may also be mutagenic [18]. 5-ASA/mesalazine is usually given as a standalone drug for treatment of IBD to avoid the side-effects associated with sulfapyridine. Despite this, oral mesalazine is not easily absorbed systemically, making SSZ the preferred drug for treatment of RA. However, the side-effects of SSZ may be poorly tolerated in RA patients and include nausea, headache, and gastrointestinal pain. More serious side-effects include anemia, kidney stones, and liver disease [19].

The antibiotic metronidazole is used in the treatment of various anaerobic bacterial infections and sometimes Crohn's disease, and is activated by the microbes that eventually become its target. The inactive drug is transported into bacterial cells and activated by nitro reductase under anaerobic or microaerophilic conditions, making it specific to low-oxygen environments such as the large intestine [20]. Activation leads to the generation of free radicals, mutagenicity and the formation of acetamide, a potentially carcinogenic by-product [21]. Metabolites of metronidazole lead to bacterial cell death and are expelled in feces with the possibility of contaminating the environment [22].

### 2.3. Immune checkpoint inhibitors and intestinal microbiota profiles

Immune checkpoint inhibitors (ICIs) are drugs that block the action of the particular inhibitory proteins that prevent T cells from killing cancer cells. For example, the combined effect of the protein PD-L1 (CD274) on tumor cells and the protein PD-1 (CD279) on T cells prevents the T cells from killing tumor cells [23]. Similarly, the protein CTLA4, which is expressed by activated T cells, acts as an “off switch” when bound to CD80 or CD86 on antigen-presenting cells [23]. These blocks to the tumoricidal activity of T cells can be overcome with ICIs and monoclonal antibodies that block the interactions of inhibitory proteins with T cells, enabling T cells to attack cancer cells.

The efficacy (as anti-cancer agents) and toxicity of ICIs with anti-PD-1, anti-PD-L-1 and anti-CTLA-4 activity has been associated with the presence or absence of particular microorganisms in the intestinal microbiota [24]–[28]. In germ-free or antibiotic-treated conventional mice, attempts to block CTLA-4 interactions with CD80/86 using anti-CTLA-4 antibodies are ineffective at activating T-cells and reducing tumor progression, suggesting that the intestinal microbiota is required for the beneficial effect of blocking CTLA-4 [28]. The introduction of *Bacteroides fragilis* to germ-free mice restored their ability to respond to anti-CTLA-4 antibodies by increasing T-cell responses close to the tumor site. The higher abundance of members from the phylum Bacteroidetes was also associated with protection from anti-CTLA-4-induced colitis, further confirming the benefits that these bacteria could have in anti-CTLA-4 antibody therapy [24]. Pre-treatment abundance of *Akkermansia muciniphila* in the intestinal microbiota of patients undergoing anti-PD-1 therapy, and co-administration of *Bifidobacterium* species with anti-PD-L-1 therapy, increased treatment efficacy via a similar mechanism to that for anti-CTLA-4 antibodies [25],[26]. It is therefore likely that the presence of particular intestinal bacteria could be helpful in increasing the efficacy of drugs targeting immune system pathways, or where stimulation of immunity is important.

### 2.4. Drug-induced changes in the intestinal microbiota

Aside from xenometabolism and the presence of treatment-enhancing bacteria that change drug bioavailability and efficacy, it is possible that drug-induced changes in the composition and function of the intestinal microbiota could also be an important mechanism influencing disease progression. One such example of this is metformin, a commonly prescribed drug for treatment of hyperglycemia in patients with type 2 diabetes (T2D). A central role for the GI-tract and/or the intestinal microbiota in metformin activity is supported by the reduced efficacy of intravenous metformin, and of oral metformin after the administration of antibiotics [29],[30]. The mechanisms involved in the glucose-lowering effect of metformin are still unclear, although its primary action is thought to be via inhibition of gluconeogenesis in the liver via activation of AMP-activated protein kinase (AMPK), which alters the expression of gluconeogenesis-associated genes [31]. Like many other chronic diseases, T2D is associated with intestinal microbial dysbiosis [32]. The beneficial effects of metformin may also be achieved via manipulation of the microbiota, for example, by enhancing the growth of mucin-degrading and probiotic *Akkermansia* species [29]. Longitudinal studies of the microbiotas of mice and humans following metformin administration showed an increase in abundance of *Akkermansia* species and other mucin-degrading taxa such as *Clostridium cocleatum* and *Bifidobacterium bifidum* which also produce beneficial SCFA [33]–[35]. Glucose tolerance was improved in mice on a high-fat diet when they were orally administered *Akkermansia* species. This suggests that increased abundance of *Akkermansia* species following metformin treatment contributes to this drug's mode of action [29]. Additionally, *Akkermansia* inhibits hyperglycemia and increases expression of peptides involved in glucose homeostasis [36].

## 3. Microbial metabolism of drugs in rheumatoid arthritis

With changes in microbial diversity and composition come changes in the metabolic capabilities of the microbiota, potentiating differences in its xenobiotic capabilities. Healthy individuals may respond uniquely to drug treatments based on the community of species present in their microbiota. When the microbiota is dysbiotic, variability in responses could increase with unknown consequences for drug efficacy. In this section we will use current knowledge to discuss the possible mechanisms for drug-microbiota interactions in the treatment of early RA with methotrexate (MTX).

RA is a chronic debilitating disease of unknown etiology in which the synovial lining of the joints becomes inflamed. Changes in the intestinal microbiota of patients with RA include an increased abundance of *Prevotella copri* during early disease, an increase in abundance of *Lactobacillus* species, and a decrease in abundance of *Bacteroides* species. However, results from different studies are inconsistent, most likely as a consequence of differences in study design and methodology (particularly sequencing and analysis), and/or the characteristics of the subject population [2]–[5]. Current treatments available for RA include a range of disease-modifying anti-rheumatic drugs (DMARDs) of which MTX is usually the first-line treatment.

### 3.1. Methotrexate action and clinical efficacy

Clinical responses to MTX are highly variable with 50% of patients achieving a 20% reduction in their swollen-and-tender-joint count (American College of Rheumatology 20; ACR20 response) and 31% achieving a good response after 3 months according to the criteria of the European League Against Rheumatism (EULAR) [37]. Despite investigations into the predictive capabilities of age, sex, BMI, smoking status, genetics and serum biomarkers, there are currently no reliable indicators of MTX treatment response [38]. Recently, differences in the composition of the intestinal microbiota before MTX treatment were shown to be correlated with clinical responses after 3 months of treatment, suggesting that the intestinal microbiota played a role in determining clinical response [4]. Additionally, compositional changes in the microbiota were induced by MTX, raising the possibility that changes in the microbiota are involved in the drug's mode of action.

**Figure 2.**
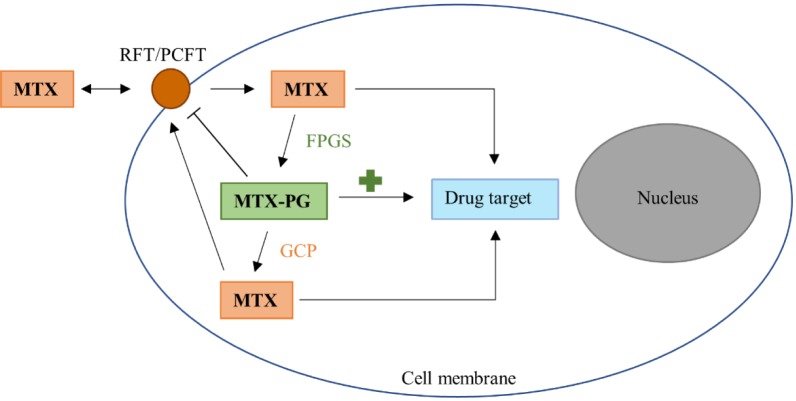
Transport and metabolism of methotrexate in humans. Methotrexate (MTX) is transported into cells via the reduced folate transporter (RFT) or proton-coupled folate transporter (PCFT). On entry, MTX is polyglutamated to MTX-PG by the enzyme folylpolyglutamate synthase (FPGS), which retains the drug inside the cell and increases its affinity for the drug target. MTX-PG may then have glutamate entities removed by glutamate carboxypeptidase (GCP), and the single-glutamate MTX may be transported out of the cell and re-enter circulation.

Once acquired into a cell, MTX is polyglutamated by folylpolyglutamate synthase (FPGS) to MTX-PG, which is thought to be the more potent than MTX itself (Figure 2). The concentration of various lengths of MTX-PGs in red blood cells is associated with treatment responses [39],[40]. After removal of the polyglutamate tail by either folate hydrolase or glutamate carboxypeptidase II, MTX-PG may then be exported. Inside the cell, MTX and MTX-PG act as competitive inhibitors of dihydrofolic reductase (DHFR), leading to a decrease in the number of metabolites required for the generation of compounds involved in DNA synthesis; this is essentially an induced folate deficiency. MTX-PG also inhibits 5-aminoimidazole-4-carboxamide ribonucleotide (AICAR) transformylase, which causes accumulating adenosine to leave the cell and bind receptors on surrounding cells, thus delivering an anti-inflammatory effect [41]. As the folate pathway is not limited to humans, it is possible that the assimilation or metabolism of MTX by intestinal bacteria could influence MTX bioavailability and efficacy.

### 3.2. Metabolism of methotrexate by folate-requiring bacteria

After internalisation via one of the various folate transporters, MTX is polyglutamated by the enzyme FPGS into a variety of MTX-PGs in humans. MTX-PG has a higher affinity for its target proteins than MTX, but also a lower affinity for folate transporters than MTX. This means that MTX-PGs are poorly transported in and out of cells. Removal of glutamate entities from MTX-PGs by carboxypeptidase reduces its efficacy as an inhibitor of DHFR. A carboxypeptidase expressed by *Pseudomonas* species, which cleaves glutamate entities from MTX (CPG2), was identified in 1967 [42]. Previous studies had demonstrated assimilation and metabolism of folic acid and its analogues by various bacteria and yeasts, including *Streptococcus faecalis*, *Enterobacter aerogenes* and *Candida tropicalis* [43]. CPG2 is now used as a rescue agent for high-dose MTX toxicity as it rapidly reduces the number of MTX-PGs in the blood, reversing the induced folate deficiency. CPG2 has orthologous enzymes in other bacterial species, such as *p*-aminobenzoyl-glutamate hydrolase (PGH) in *E. coli*, which can also act on MTX [44]. These enzymes have essential functions in folate-requiring bacteria which could potentially assimilate and metabolize MTX, thereby altering its efficacy. In addition to removal of glutamate via carboxypeptidase, some intestinal bacteria have the capacity to add glutamate to MTX via FPGS-like enzymes. In a study investigating the impact of MTX on the metabolism of a panel of 25 intestinal microbes, at least two species could metabolize MTX to MTX-PG [45].

As MTX-PG is not transported as easily as MTX, is it unlikely that intestinal bacteria-generated MTX-PG would affect treatment outcomes because the MTX-PG would likely be retained by the cell or converted back to MTX prior to export. Additionally, removal of glutamate entities from MTX via, for instance, *Pseudomonas*' CPG2, would be unlikely to decrease efficacy of MTX as it would not affect post-absorption formation of MTX-PGs. However, switching from oral to parenteral administration of MTX leads to a significant increase in long and very long-chain MTX-PGs, suggesting that the oral route may inhibit formation of MTX-PGs, possibly via assimilation of MTX by the microbiota [46].

### 3.3. Intestinal microbiota profiles and methotrexate efficacy

To date only one study has investigated the influence of the microbiota on determining treatment outcomes in response to MTX [4]. Shotgun metagenomics was used to characterize the salivary, dental, and fecal microbiotas of treatment-naïve RA patients before and after the use of MTX in combination with other DMARDs [4]. Differentially-abundant species were identified in baseline samples from patients that were responding well, moderately or not at all (based on DAS28 reduction) and it was found that MTX changed the composition of the fecal microbiota. In particular, four RA-enriched unclassifiable taxa and one species that was most closely related to *Enterococcus faecium*, decreased in abundance following treatment, suggesting that MTX may act either by decreasing the abundance of disease-associated bacteria or by restoring a “healthy” microbiota. Notably, there were more changes to the composition of the dental and salivary microbiota than to the composition of the fecal microbiota; there were increases in several healthy control-associated taxa in the dental and salivary microbiota following treatment, suggesting that these microenvironments are more susceptible to MTX-induced changes and may also be involved in treatment responses. This study also generated predictive models using the microbiota data collected, which were able to differentiate between good and poor responders. These observations provide evidence that microbiota-based variables may be important in determining whether an RA patient responds well or poorly to treatment with MTX, although the underlying mechanisms remain unknown.

MTX treatment failure in RA patients may lead to the use of other DMARDs or biological treatments such as TNF inhibitors, which also carry a variable treatment response and lack reliable predictive factors [38]. No studies have specifically linked microbiota profiles with treatment responses to TNF inhibitors in RA patients. However, investigations with IBD patients suggest partial recovery of microbial dysbiosis is induced by TNF inhibitors, and response-associated baseline microbiota profiles have been identified in patients with spondyloarthritis [47],[48]. The possibility of using microbiota signatures to guide treatment (TNF inhibitors) decisions, and the effect of various lifestyle factors on treatment outcomes, is currently being investigated in a prospective cohort study in Denmark [49]. In this study the collection of several lifestyle and dietary variables alongside microbiota data will allow both identification of predictive markers in the microbiota, and also the associated lifestyle factors that can be modified to improve treatment efficacy. This is an important step forward in translating microbiota studies into the clinical management of chronic inflammatory diseases such as RA.

## 4. Conclusions

Our understanding of the various mechanisms by which the human intestinal microbiota may contribute to treatment responses is expanding. However, more mechanistic studies are needed to unravel the ways in which bacteria and other microbes interact with drugs to influence their bioavailability and efficacy. Associations between the microbiota and treatment responses (of which several are known) need to be followed up with mechanistic *in vitro* and *in vivo* studies to identify the specific taxa involved and define their interactions with particular drugs, the environment, and the host. From a clinical perspective, elucidation of these mechanisms could greatly improve personalized medicine because it would reduce the costs and harm to patients that is caused by “trial and error” use of drugs (such as MTX for RA patients) with highly variable treatment responses. Using fecal samples as a surrogate for the intestinal microbiota, it is possible to profile and identify the composition of the microbiota prior to treatment which could inform clinical decisions on which treatment and/or adjuvants to use, thus improving clinical response and patient quality of life. Known organisms associated with good or poor responses could be targeted. For example, prebiotics, probiotics, fecal microbiota transplant, or personalized nutrition could introduce and maintain beneficial microbes, or antibiotics could be used selectively to remove unwanted microbial populations.
